# The foramen magnum in scaphocephaly

**DOI:** 10.1007/s00381-022-05624-2

**Published:** 2022-08-05

**Authors:** Tymon Skadorwa, Olga Wierzbieniec

**Affiliations:** 1 Department of Pediatric Neurosurgery, Bogdanowicz Memorial Hospital for Children, 4/24 Nieklanska St, 03924 Warsaw, Poland; 2 Department of Descriptive and Clinical Anatomy, The Medical University of Warsaw, 5 Chalubinskiego St, 02004 Warsaw, Poland

**Keywords:** Foramen magnum, Craniosynostoses, Children, Scaphocephaly, Occipital bone, Skull base

## Abstract

**Purpose:**

The foramen magnum (FM) presents various alterations in craniosynostoses, such as brachycephaly or Crouzon syndrome. However, to date, no study has been devoted to its morphology and morphometry in scaphocephaly, which is the most common of cranial deformities resulting from premature fusion of cranial sutures.

**Methods:**

We assessed the morphology and morphometry of FM using preoperative thin-cut CT scans of 107 children with non-syndromic sagittal craniosynostosis aged 1–12 months (mean age 5.38 months). A series of sagittal and transverse dimensions were taken and the FM area was calculated in each case. Obtained data were compared to the age-matched control group of 101 normocephalic children.

**Results:**

Dolichotrematous type of FM was dominant in the scaphocephaly group and observed in 63/107 cases (58.9%). The mean FM area in the scaphocephaly group was 519.64 mm^2^ and was significantly smaller compared to the control group (*p* = 0.0011). The transverse diameter and anterior sagittal diameter were also significantly smaller (*p* = 0.0112 and *p* = 0.0003, respectively).

**Conclusion:**

The area of FM in scaphocephaly is smaller compared to normal individuals. This is associated with a significant reduction of the width of FM in children with sagittal craniosynostosis. FM in scaphocephaly is larger than in other reported series of children with brachycephaly or Crouzon syndrome.

## Background

The development of the skull base has gained a particular interest in patients with craniosynostosis [1–3]. In recent years, the morphology of the foramen magnum (FM) has been frequently studied not only in children with syndromic and non-syndromic craniosynostosis [4, 5], but also in people not burdened with congenital malformations of the skull [6, 7]. These studies allowed for the description of the morphology of FM in anatomical and developmental terms, in particular in conjunction with clinical data such as the development of hydrocephalus or the position of cerebellar tonsils and the presence of Chiari malformation [8–10].

Particularly important data on the morphology and dimensions of FM were obtained from the studies describing patients treated for syndromic craniosynostoses, such as Crouzon, Apert, Pfeiffer, Muenke, or Saethre-Chotzen syndromes, or with achondroplasia [11–14]. These works described FM in brachycephalic skulls, but the literature still lacks data on the morphology of FM in scaphocephaly, resulting from the premature fusion of sagittal suture [15].

The prevalence of non-syndromic sagittal synostosis is estimated for 1:5000 live births and the reported M:F sex ratio is 3:1 [16]. The disease is considered to have a genetic background as autosomal dominant with a penetration rate estimated at 36% [17]. Children with sagittal synostosis have normal IQ; however, in 7–37%, they present a delay in speech development and other cognitive deficits [18]. The incidence of hydrocephalus in scaphocephaly is low and amounts to 0.3%, and the incidence of Chiari malformation is estimated at 3–15% [19, 20].

To date, most of the published papers aimed to describe FM in patients with syndromic craniosynostoses. The literature still lacks data concerning the FM in the most common cranial deformity. Our goal was to study the morphology and morphometry of FM in scaphocephaly and to place them in a developmental context.

## Patients and methods

### Population

The group of patients with scaphocephaly (non-syndromic sagittal craniosynostosis) aged 1–12 months included 107 children treated in our institution between 2010 and 2020. All patients presented a sagittal synostosis with respective cranial deformation (scaphocephaly with one or combination of the following: retroorbital depression, saddle deformation, frontal or occipital bossing). All patients had preoperative thin-cut CT scans. The mean age of the scaphocephaly group was 5.38 months, the median age was 4.97 months, and the standard deviation was 2.47. The sex ratio was 2.96.

An age-matched control group was designed (mean age 6.30 months, median age 6.03 months, standard deviation 2.70, sex ratio 2.88). The control group included 101 infants with CT scans performed due to a head injury (extra- and intraaxial lesions or post-operative changes were not included in the study).

The study protocol has been approved by the local Bioethics Committee (decision number AKBE/110/2021).

### Morphologic assessment

The morphology of FM was investigated using preoperative thin-cut CT scans provided with Siemens Somatom Emotion with parameters: slice thickness 0.5 mm; the exposition was performed with source voltage 270 kV and current of 100 mA. All scans were analyzed with RadiAnt DICOM Viewer PL version 2021.2.2 (64 bit). The shape of FM was assessed according to the classification proposed by Richards and Jabbour [21]. Each FM was also classified as dolichotreamatous, mesotrematous, or brachytrematous, as used by Burdan et al. [6, 22].

### Morphometric analysis

**Fig. 1:**
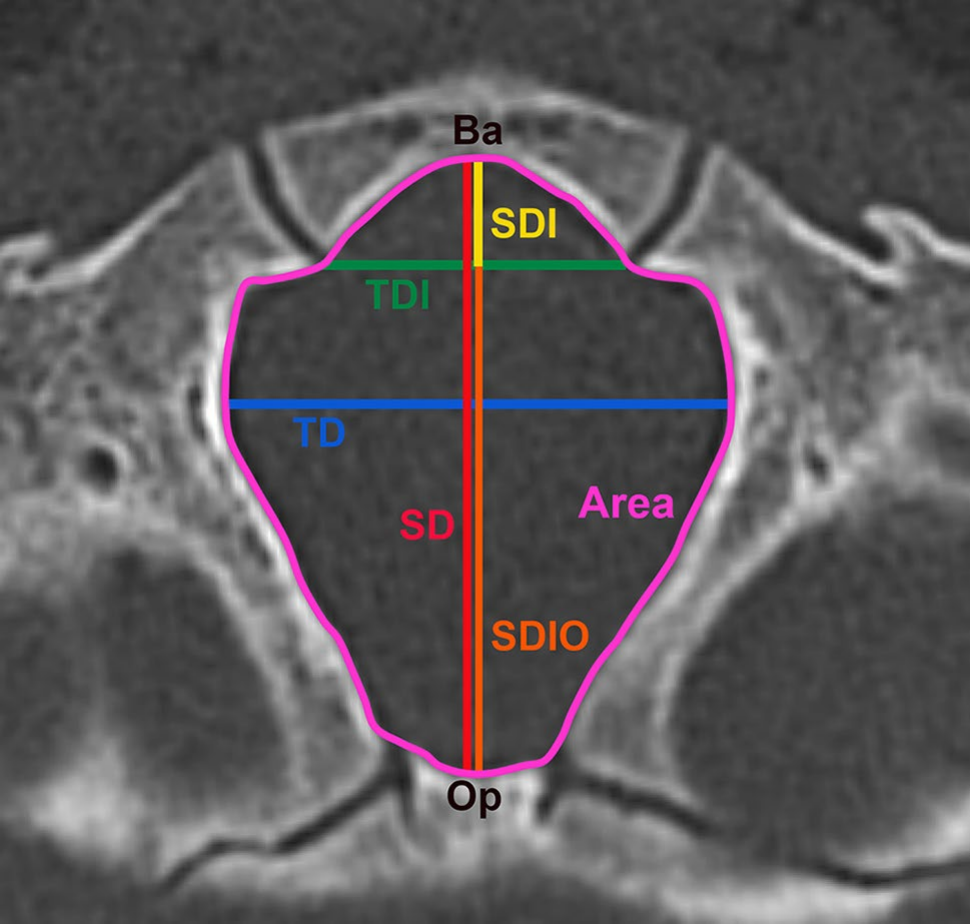
Measurements taken at the level of FM (CT scan, axial cut); Ba, basion; Op, opisthion; area—surface within the purple line

The milimetric data were obtained from preoperative CT scans. Six measurements of FM were performed (Fig. 1), according to the study of Coll et al. [11] and included:

- Area: the foramen magnum area (mm^2^)
- SD (sagittal diameter): the maximal dimension from basion to opisthion (mm)
- SDI (anterior sagittal diameter): from basion to anterior bi-interoccipital synchondrosis (mm)
- SDIO (posterior sagittal diameter): from anterior bi-interoccipital to opisthion (mm)
- TD (transverse diameter): the foramen magnum maximal transverse dimension (mm)
- TDI (anterior transverse diameter): the maximal dimension between interoccipital synchondroses (mm)

### Radiologic features of scaphocephaly

Each patient with scaphocephaly was assessed for the widening of the subarachnoid space and the ventriculomegaly, as proposed in the paper by Diab et al. [23]. The subarachnoid space was assessed in frontal regions and in the anterior portion of the longitudinal cerebral (interhemispheric) fissure (in both regions the width > 5 mm was considered as widened). Ventriculomegaly was calculated by Evans index > 0.3, as proposed by Diab et al. [23].

The scaphocephaly patients were also compared for differences in skull shape. A classification including 5 distinct types was used (dolichocephaly, leptocephaly, clinocephaly, bathrocephaly, sphenocephaly), as in the paper by Di Rocco et al. [24]. The parameters of FM were compared accordingly.

### Statistical analysis

The measured parameters were compared with the directional non-parametric Mann–Whitney-Wilcoxon rank-sum test using z-scores. We used this method for two reasons: (1) the pattern of head growth is age specific and does not follow a linear model and (2) we aimed the measured parameters and statistical method to be comparable with previously published studies of FM morphometry in other types of craniosynostosis [5, 11]. The alternative hypothesis (control group measurements higher than in scaphocephaly group) was accepted if the results were considered significant (*p* < 0.05).

A graphical analysis including Loess non-parametric regression for each parameter was used after the method described in the paper of Coll et al. [11]. Best-fit curves extrapolating the change of measured dimensions during the first postnatal year were calculated on the scatterplot of each measured dimension against age using 0.5 as the smoothing parameter.

The statistical analysis was performed with TIBCO Data Science/Statistica software by StatSoft Europe, version 13.3 PL for Microsoft Windows 10 Pro.

## Results

### Morphologic assessment

The FM varied in shape. An advantage of the longitudinal dimensions was observed in all cases, resulting in oval shapes recognized in both groups. Dolichotrematous type dominated in both the scaphocephaly group (63/107 cases, 58.9%) and the control group (53/101 cases, 52.4%). The brachytrematous type was found in 23/107 scaphocephaly cases (21.5%) and in 29/101 controls (28.7%). The mesotrematous type was the least frequent (21/107 cases (19.6%) and 19/101 cases (18.8%), respectively).

In the studied population, most of the types distinguished by Richards and Jabbour [21] were found. The most common shape in the scaphocephaly group was heart-like FM (84/107 cases, 78.5%), followed by bi-rounded oval (10/107, 9.3%) and dorsally convergent oval (8/107, 7.5%). The same type (heart like) dominated in the control group (32/101 cases, 31.7%). Other types found in this group were the variations of oval FM: two semicircle (31/101, 30.7%), dorsally convergent oval (26/101, 25.7%), bi-rounded oval (11/101, 10.9%), and bi-pointed oval (1/101, 1%).

### Measured parameters of FM

Basic statistics calculated for each parameter are presented in Table 1. No significant differences between boys and girls were noted.

**Table 1:** Basic statistics of measured parameters; *sd*, standard deviation

	Scaphocephaly		Control		*p*-value
	Mean value ± sd	Min–max	Mean value ± sd	Min–max	
Area (mm^2^)	519.60 ± 81.03	354.20–703.30	563.25 ± 85.09	380.20–757.30	0.0011
SD (mm)	30.77 ± 2.65	24.30–38.10	31.39 ± 2.72	24.60–38.20	0.1066
SDI (mm)	5.36 ± 0.77	3.07–7.32	5.75 ± 0.72	3.93–7.72	0.0003
SDIO (mm)	25.41 ± 2.47	19.01–31.18	25.64 ± 2.53	19.99–31.69	0.5833
TD (mm)	24.92 ± 2.16	19.20–30.60	25.67 ± 2.02	20.00–30.60	0.0112
TDI (mm)	14.68 ± 1.54	11.60–18.60	14.49 ± 1.54	10.30–17.40	0.6186

### Foramen magnum area

**Fig. 2:**
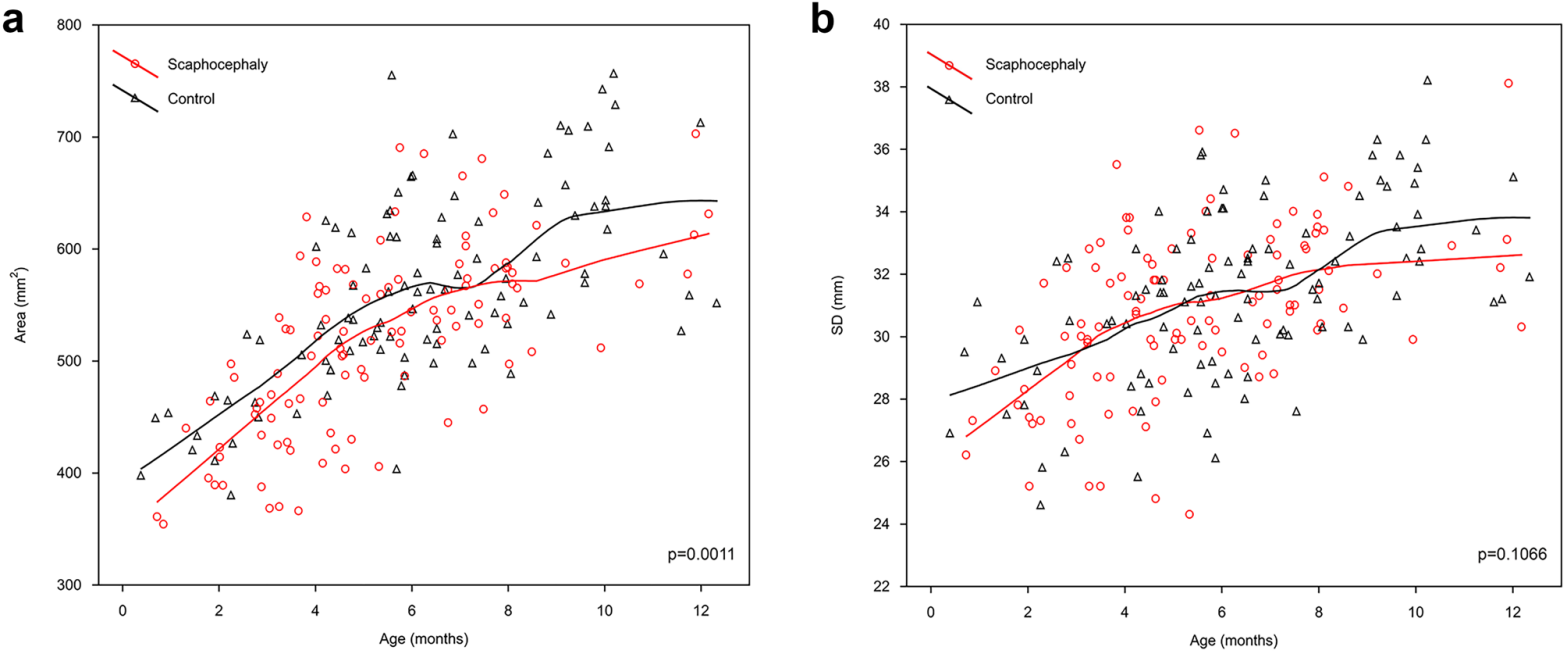
The distribution of foramen magnum area **a** and SD **b** by age

We found a significant difference in the FM area when comparing children with the scaphocephaly and control group (*p* = 0.0011, *z* score = 3.268). The mean area of FM in scaphocephaly was 519.64 mm^2^. The Loess curve of the FM area for the scaphocephaly group was situated below the curve of the control group with an intersection of two lines at the age of 7 months. The maximal rate of increase of the FM area for both groups was observed during the first 6 months after delivery. In both groups, the maximal value of the FM area was reached between the 11th and 12th months (Fig. 2a).

### Sagittal diameter (SD), anterior sagittal diameter (SDI), and posterior sagittal diameter (SDIO)

The sagittal diameter of FM (SD) did not significantly differ between the groups (*p* = 0.1066, *z* = 1.613) (Fig. 2b). The growth rate in the scaphocephaly group was high from birth to 4th month and stable afterwards, while in the control group it revealed two phases: a stable phase from birth to 6th month and a more dynamic phase from 7 to 9th month. These two periods of increased growth in the control group were separated by a plateau at the level of 30–31 mm. Between the 3rd and 7th month, the sagittal diameter in scaphocephaly was comparable to that of the control group. After the 7th month, the curve of the control group intersected the line of the scaphocephaly group taking advantage of values that remained higher till the end of the first year.

**Fig. 3:**
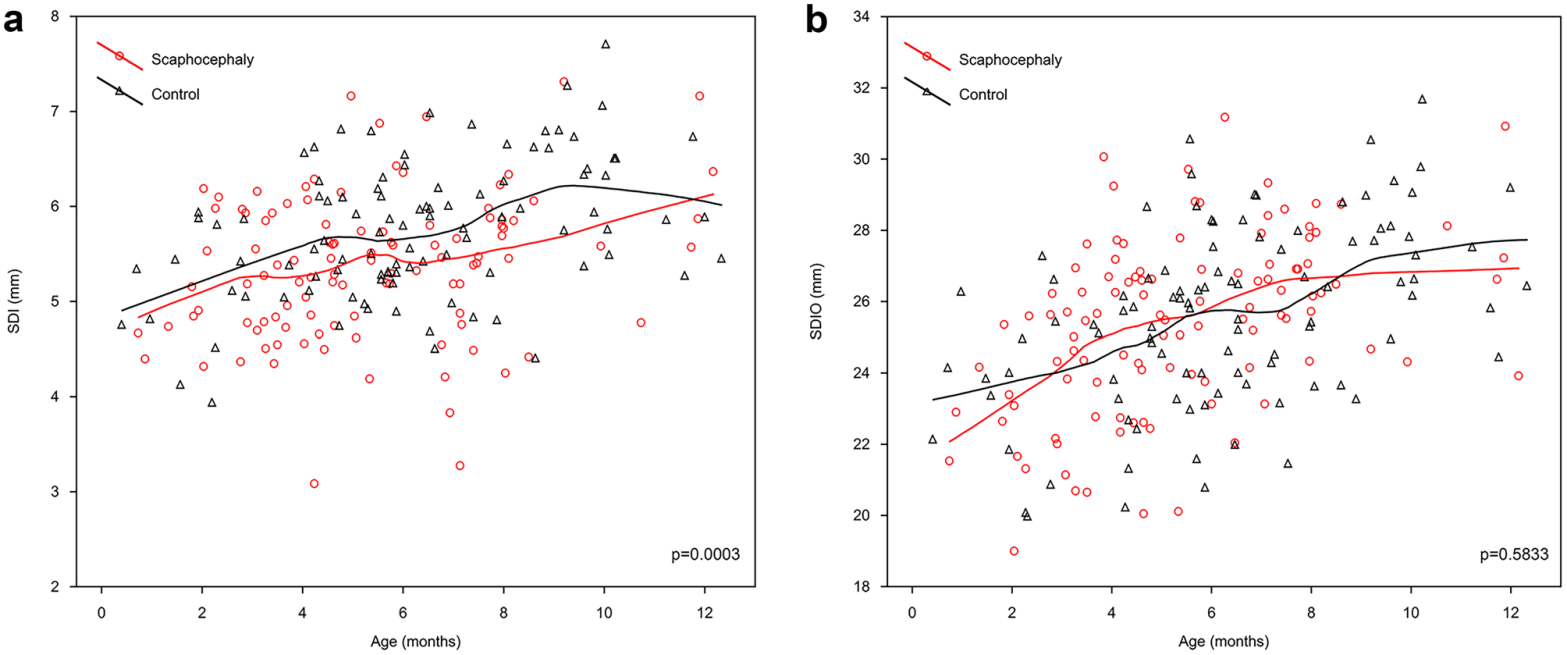
The distribution of SDI **a** and SDIO **b** by age

The anterior sagittal diameter (SDI), representing the length of the ventral portion of FM, was significantly smaller in the scaphocephaly group compared to the control group (*p* = 0.0003, *z* = 3.571). The growth rate was rising constantly but the curve of the scaphocephaly group was below that of the control group through the first 12 months. Interestingly, both groups had a short decrease between the 5th and 6th month, but from this point the growth rate of the scaphocephaly group was constantly rising, while the one of the control group reached a peak at the 9th month and then started to decrease (Fig. 3a).

The posterior sagittal diameter (SDIO) difference was not statistically significant between the scaphocephaly and control groups (*p* = 0.5833, *z* = 0.548). The Loess curve of scaphocephalic children was situated over the line of the control group from the 3rd to the 9th month and presented a more constant growth rate, with a tendency to stabilize after the 8th month. The growth rate of the control group was more dynamic after the 7th month and it tended to stabilize around the 10th month (Fig. 3b).

### Transverse diameter (TD) and anterior transverse diameter (TDI)

**Fig. 4:**
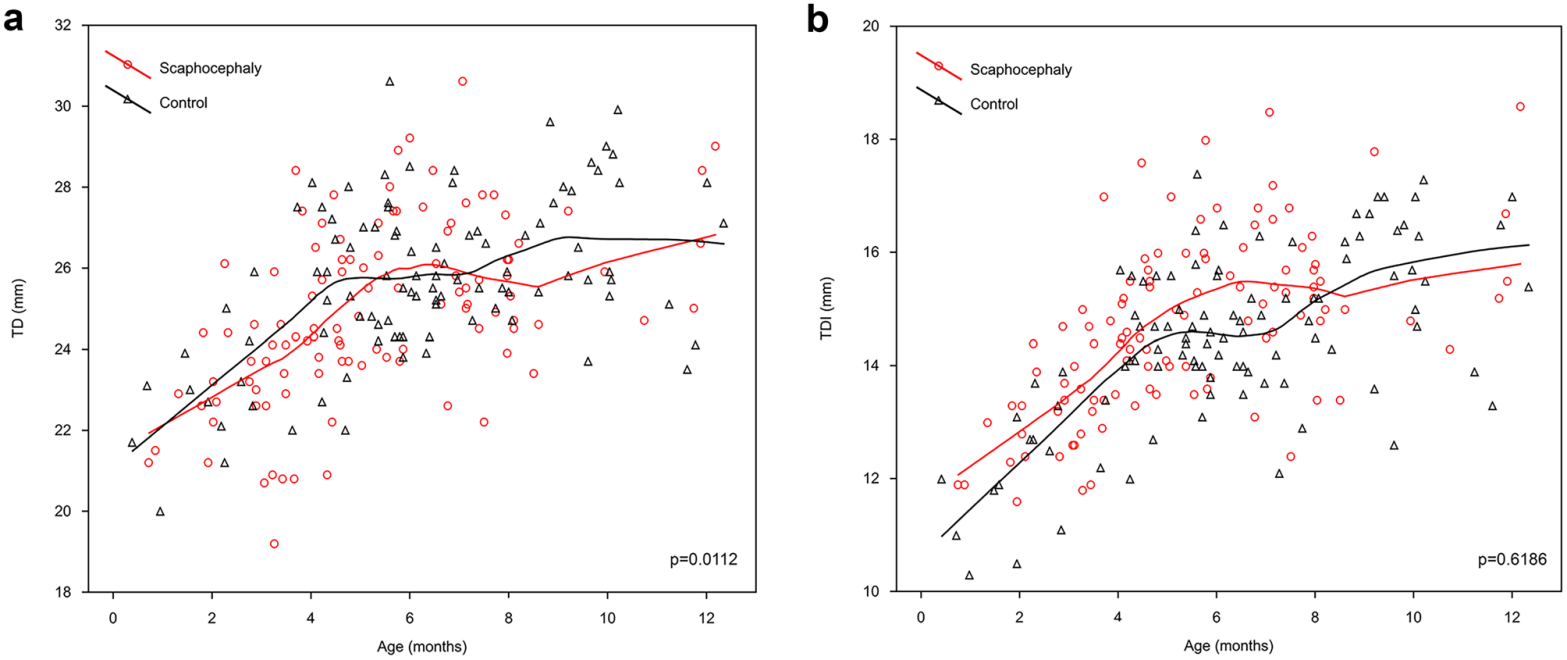
The distribution of TD **a** and TDI **b** by age

A statistically significant difference in transverse diameter (TD) was noted in scaphocephaly group (*p* = 0.0112, *z* = 2.535). The extrapolation of data revealed two phases of the rapid growth of this parameter in scaphocephalic children: the first phase was from birth to the 6th month and the second began after the 8th–9th month. At 6 months, the values of TD in the scaphocephaly group were comparable to those of the control group. Despite these observations, the Loess curve of the scaphocephaly group was mostly situated below the curve of the control group through the first 12 months. The lines of both groups intersected three times: at 5th, 7th, and 12th months showing that the transverse diameter of FM in scaphocephaly undergoes a dynamic but differential growth pattern. The highest values in the control group were observed in the 9th month; then, the growth tended to slow down, while the one of the scaphocephaly group presented a tendency to increase from the 9th month (Fig. 4a).

The analysis of anterior transverse diameter (TDI) showed no significant difference between the groups (*p* = 0.6186, *z* = 0.498). However, contrarily to TD, the Loess curve of the scaphocephaly group was over the line of the control group through the first 8 months. The growth in the control group was more dynamic after the 7th month and the curve intersected the one of scaphocephaly after the 8th month (Fig. 4b).

### Growth rates in the first 12 postnatal months

The analysis of the growth pattern of all measured parameters during the first year showed that the growth rate in scaphocephaly was considerably higher than in the control group in terms of area, SDI, and TD.

The growth of FM area in children with scaphocephaly was higher than that of the control group (22.2 vs. 20.0 mm^2^/month, respectively). Also, the total increase for the area was higher in the scaphocephaly group (163 vs. 146%, respectively). Despite this fact, the absolute values of the FM area in the scaphocephaly group were significantly lower than these of the control group.

When comparing the growth rates of sagittal diameters (SD, SDI, and SDIO), we found that the scaphocephaly group presented comparable growth rates to the controls. In the first 12 months, the growth of SD in the control group (0.53 mm/month) was almost equal to that of the scaphocephaly group (0.54 mm/month) with a slightly lower total increase (118 vs. 122%, respectively). For the SDI, the growth rate in the control group was 0.13 mm/month (126% increase in SDI) while in the scaphocephaly group it was 0.15 mm/month (136% SDI increase). Comparing the SDIO, the control group revealed an equal growth compared to the scaphocephaly group (0.39 mm/month in both groups). The total increases in SDIO were also comparable for both groups (116 and 119%).

In the first 12 months, the growth rate of TD was 0.49 mm/month (125% of the total increase) in the scaphocephaly group and 0.38 mm/month (117% of the total increase) in the control group. The scaphocephaly group had a comparable growth rate and a total increase of TDI (0.38 mm/month and 135%, respectively) when compared to the control group (0.39 mm/month and 134%, respectively).

### Radiologic features of scaphocephaly

In the scaphocephaly group, a widened subarachnoid space was observed in 96/107 cases (89.7%) and ventriculomegaly in 70/107 cases (65.4%). Despite high occurrence, none of these features significantly affected the parameters of FM. Significant differences, however, were noted for FM area and transverse diameters (TD and TDI) between the types of skull shapes. The smallest FM area was observed in sphenocephaly (mean value 487.00 mm^2^, *p* = 0.0035) and the greatest in dolichocephaly (mean value 565.90 mm^2^). Sphenocephalic patients presented also the smallest mean values of TD (24.2 mm, *p* = 0.0223) and TDI (14.1 mm, *p* = 0.0125). The results of the analysis are included in Supplementary Material.

## Discussion

The ontogenesis of FM has been described from various perspectives [25, 26]. Embryologic data explain how the sclerotomic primordia, involved in the formation of FM, derive from the caudal part of the 4th and rostral part of the 5th somites [27]. During resegmentation, they form the proatlas sclerotome, which gives origin to three elements of the occipital bone: basiocciput, exoocciput, and supraocciput, separated by the anterior and posterior interoccipital synchondroses and undergoing the intramembranous and endochondral ossification [26, 28, 29].

The area of FM is developmentally divided into two parts: ventral (anterior) and dorsal (posterior), that present distinct growth patterns [21]. According to Richards and Jabbour, the dorsal part increases slower than the ventral in the first 6 months after birth and its growth rate constantly decreases by the 12th month, while the ventral part continues to grow at a high rate until the age of 1 year. In the paper by Di Rocco et al., analyzing the growth of FM in brachycephalic skulls, the authors suggest that in the first six postnatal months the increase of breadth of FM dominates over the increase of its length [5]. Our data is consistent with the observations of Coll et al.—in our population, the growth of SDIO was faster than this of SDI.

The developmental pattern allows the formation of various anatomical types and shapes of FM. Richards and Jabbour distinguished eight distinct anatomical variants, based on the relations between the sagittal and transverse diameters of FM [21]. This relation was studied in the paper of Burdan et al. [6], where three types of FM, analogous to classic anthropologic indices [22], have been used for analysis. In our group of scaphocephalic children, the dolichotrematous type of FM was found the most common, followed by brachytrematous and mesotrematous types, while in the cohort of Burdan et al. the brachytrematous type was the most frequent. It is an interesting finding, regarding the comparable growth rates of sagittal dimensions in scaphocephalic skulls in relation to normal skulls and highlighting the observed difference in TD. In both cohorts, the one of Burdan et al. and ours, the mesotrematous type was the least common and accounted for 19.2% and 19.6%, respectively.

Other authors have also investigated the shape of FM [7], but used slightly different criteria; therefore, our analysis of FM shape follows the classification by Richards and Jabbour, as a reference. In our population, the heart-like shape dominated in the scaphocephaly group, while the dorsally convergent oval shape was the most common in the control group. This classification is also mentioned in the study of Burdan et al., which is important for us, as it refers to the Eastern European population [6]. However, contrarily to that paper, in our population, the differences in length and width of FM between males and females were not significant. It should be noted that the measurements in the mentioned paper were taken on adult material.

### Comparative analysis of FM in the scaphocephaly vs. control group

The comparison of the measurements taken in the scaphocephaly group with those obtained in the control group revealed a number of differences. The most important of these is that in general the FM was smaller in scaphocephaly than in the control group.

Our results showed that the area of FM in scaphocephaly was significantly smaller compared to the controls (*p* = 0.0011). This may be associated with an observed significant reduction of FM width in this group. This is an intriguing observation, as the growth rate of TD was higher in children with scaphocephaly. Meanwhile, all sagittal dimensions in this group presented comparable growth rates to the controls, but the only parameter having significantly smaller values was the SDI. This finding is in line with the theory that the dorsal part of FM develops faster than the ventral part, but it seems that scaphocephalic skulls follow yet another model of development where the growth in the sagittal axis of FM is comparable to normal but in the transverse axis of FM it is faster, even though the transverse diameter does not statistically reach the level of healthy individuals. This alteration combined with a comparable length of FM with the control group probably contributes to a smaller area of FM in scaphocephaly and perhaps accounted for the observed domination of heart-like shape.

### Scaphocephaly vs. other reported skull deformities

Our results differ from other published series including patients with brachycephaly (isolated and syndromic) and Crouzon syndrome (Table 2). The FM area in scaphocephaly was greater when compared to other craniosynostoses. The mean value of 519.64 mm^2^ in our series would place the scaphocephaly above the reported brachycephaly (460.84 mm^2^) and Crouzon (471.18 mm^2^) groups [5, 11]. Comparing the SD and SDIO, scaphocephaly presented greater values than two other series, but in terms of SDI it was placed between brachycephaly and Crouzon syndrome cases. For the TD, Crouzon syndrome patients had the smallest values and scaphocephalic patients the greatest, but for the TDI, the smallest values were observed in brachycephaly, followed by scaphocephaly and Crouzon syndrome. It is not surprising that the FM dimensions in brachycephaly patients were always below the scaphocephaly values, but the differences in SDI and TDI between scaphocephaly and Crouzon groups reflect a distinct pattern of FM growth in the latter with greater ventral part of FM when compared to other craniosynostoses. The comparison between these three series may suggest some similarities in terms of FM growth between brachycephaly and scaphocephaly, and a distinct pattern in Crouzon syndrome, which is probably related to the multisuture type of craniosynostosis.

**Table 2:** The comparison of mean values of measured parameters between our study and other series of craniosynostoses [5, 11]

	Number of cases	Area (mm^2^)	SD (mm)	SDI (mm)	SDIO (mm)	TD (mm)	TDI (mm)
Brachycephaly group [5]	40	460.84	29.68	5.13	24.55	23.39	13.16
Crouzon syndrome group [11]	21	471.18	28.86	6.04	22.82	22.76	15.07
Scaphocephaly group (this study)	107	519.60	30.77	5.36	25.41	24.92	14.68
Control group (this study)	101	563.25	31.39	5.75	25.64	25.67	14.49

### Limitations of the study

The main limitation of this study is related to the measurement protocol adapted from previous studies [5, 11]. We aimed the dimensions and statistical method to be comparable with other published data. A further limitation may come from the fact that the age of the studied population was limited to 12 months, which prevents drawing conclusions about the further development of FM.

## Conclusions

To the best of our knowledge, this study is the first to describe FM in children with scaphocephaly. After the studies reporting the dimensions of FM in brachycephaly and Crouzon syndrome, we could compare scaphocephaly with other craniosynostoses. The area of FM in scaphocephaly is significantly smaller compared to normocephalic individuals. This is associated with a significant reduction of the width of FM in children with sagittal craniosynostosis. The dimensions of FM in scaphocephaly were always greater when compared to reported brachycephaly patients but the ventral portion was smaller than in children with Crouzon syndrome.

## Data Availability

The data belong to Bogdanowicz Memorial Hospital for Children in Warsaw and are not available to share unless in the form included in the manuscript and supplementary materials.

## References

[1] Coll G, Lemaire JJ, Di Rocco F, Barthélémy I, Garcier JM, De Schlichting E, Sakka L (2016). Human foramen magnum area and posterior cranial fossa volume growth in relation to cranial base synchondrosis closure in the course of child development. Neurosurgery.

[2] Goodrich JT (2005). Skull base growth in craniosynostosis. Childs Nerv Syst.

[3] Skrzat J, Stepańczak B, Walocha J (2014). The scaphocephalic skull of an adult male. Folia Morphol (Warsz).

[4] Assadsangabi R, Hajmomenian M, Bilaniuk LT, Vossough A (2015). Morphology of the foramen magnum in syndromic and non-syndromic brachycephaly. Childs Nerv Syst.

[5] Di Rocco F, Dubravova D, Ziyadeh J, Sainte-Rose C, Collet C, Arnaud E (2014). The foramen magnum in isolated and syndromic brachycephaly. Childs Nerv Syst.

[6] Burdan F, Szumiło J, Walocha J, Klepacz L, Madej B, Dworzański W, Klepacz R, Dworzańska A, Czekajska-Chehab E, Drop A (2012). Morphology of the foramen magnum in young Eastern European adults. Folia Morphol (Warsz).

[7] Ulcay T, Kamaşak B, Görgülü Ö, Uzun A, Aycan K (2022). A golden ratio for foramen magnum: an anatomical pilot study. Folia Morphol (Warsz).

[8] den Ottelander BK, Dremmen MHG, de Planque CA, van der Oest MJW, Mathijssen IMJ, van Veelen MLC (2022). Does the association between abnormal anatomy of the skull base and cerebellar tonsillar position also exist in syndromic craniosynostosis?. J Plast Reconstr Aesthet Surg.

[9] Rijken BFM, Lequin MH, Van Veelen MLC, de Rooi J, Mathijssen IMJ (2015) The formation of the foramen magnum and its role in developing ventriculomegaly and Chiari I malformation in children with craniosynostosis syndromes. J Craniomaxillofac Surg 43(7):1042–1048. 10.1016/j.jcms.2015.04.025 10.1016/j.jcms.2015.04.025 26051848

[10] Sgouros S, Kountouri M, Natarajan K (2007). Skull base growth in children with Chiari malformation Type I. J Neurosurg.

[11] Coll G, Arnaud E, Selek L, Brunelle F, Sainte-Rose C, Collet C, Di Rocco F (2012). The growth of the foramen magnum in Crouzon syndrome. Childs Nerv Syst.

[12] Hecht JT, Nelson FW, Butler IJ, Horton WA, Scott CI, Wassman ER, Mehringer CM, Rimoin DR, Pauli RM (1985). Computerized tomography of the foramen magnum: achondroplastic values compared to normal standards. Am J Med Genet.

[13] Reynolds KK, Modaff P, Pauli RM (2001). Absence of correlation between infantile hypotonia and foramen magnum size in achondroplasia. Am J Med Genet.

[14] Rijken BFM, Lequin MH, de Rooi J, van Veelen MLC, Mathijssen IMJ (2013). Foramen magnum size and involvement of its intraoccipital synchondroses in Crouzon syndrome. Plast Reconstr Surg.

[15] Kajdic N, Spazzapan P, Velnar T (2018) Craniosynostosis – recognition, clinical characteristics, and treatment. Bosn J Basic Med Sci 18(2):110–116. 10.17305/bjbms.2017.2083 10.17305/bjbms.2017.2083 PMC5988529 28623672

[16] Rogers GF (2011) Deformational plagiocephaly, brachycephaly, and scaphocephaly. Part I: terminology, diagnosis, and etiopathogenesis. J Craniofac Surg 22(1):9–16. 10.1097/SCS.0b013e3181f6c313 10.1097/SCS.0b013e3181f6c313 21187783

[17] Lajeunie E, Le Merrer M, Bonaiti-Pellie C, Marchac D, Renier D (1996). Genetic study of scaphocephaly. Am J Gen Med.

[18] Osborn AJ, Roberts RM, Dorstyn DS, Grave BG, David DJ (2021). JAMA Netw Open.

[19] Cinalli G, Sainte-Rose C, Kollar EM, Zerah M, Brunelle F, Chumas P, Arnaud E, Marchac D, Renier D (1998). Hydrocephalus and craniosynostosis. J Neurosurg.

[20] Strahle J, Muraszko KM, Buchman SR, Kapurch J, Garton HJL, Maher CO (2011). Chiari malformation associated with craniosynostosis. Neurosurg Focus.

[21] Richards GD, Jabbour RS (2011). Foramen magnum ontogeny in Homo sapiens: a functional matrix perspective. Anat Rec (Hoboken).

[22] Martin R, Saller K (1959). Lehrbuch de Anthropologie.

[23] Diab J, Flapper W, Grave B, Abou-Hamden A, Anderson P, Moore M (2022). The many faces of sagittal synostosis: a novel classification and approach to diagnosis. J Craniofacial Surg.

[24] Di Rocco F, Gleizal A, Szathmari A, Beuriat PA, Paulus C, Mottolese C (2019). Sagittal suture craniosynostosis or craniosynostoses? The heterogeneity of the most common premature fusion of the cranial sutures. Neurochirurgie.

[25] Bernard S, Loukas M, Rizk E, Oskouian RJ, Delashaw J, Tubbs RS (2015) The human occipital bone: review and update on its embryology and molecular development. Childs Nerv Syst 31(12):2217–2223. 10.1007/s00381-015-2870-8 10.1007/s00381-015-2870-8 26280629

[26] Jin SW, Sim KB, Kim SD (2016) Development and growth of the normal cranial vault: an embryologic review. J Korean Neurosurg Soc 59(3):192–196. 10.3340/jkns.2016.59.3.192 10.3340/jkns.2016.59.3.192 PMC4877539 27226848

[27] Pang D, Thompson DNP (2011) Embryology and bony malformations of the craniovertebral junction. Childs Nerv Syst 27(4):523–564. 10.1007/s00381-010-1358-9 10.1007/s00381-010-1358-9 PMC3055990 21193993

[28] Couly GF, Coltey PM, Le Douarin NM (1993). The triple origin of skull in higher vertebrates: a study in quail–chick chimeras. Development.

[29] Tubbs RS, Griessenauer CJ, Loukas M, Shoja MM, Cohen-Gadol AA (2010) Morphometric analysis of the foramen magnum: an anatomic study. Neurosurgery 66(2):385–388. 10.1227/01.NEU.0000363407.78399.BA 10.1227/01.NEU.0000363407.78399.BA 20087140

